# Red‐winged blackbirds nesting nearer to yellow warbler and conspecific nests experience less brood parasitism

**DOI:** 10.1002/ece3.9818

**Published:** 2023-02-09

**Authors:** Shelby L. Lawson, Janice K. Enos, Sharon A. Gill, Mark E. Hauber

**Affiliations:** ^1^ Department of Evolution, Ecology, and Behavior University of Illinois Urbana‐Champaign Urbana Illinois USA; ^2^ Illinois Natural History Survey, Prairie Research Institute University of Illinois at Urbana‐Champaign Champaign Illinois USA; ^3^ Department of Biological Sciences Western Michigan University Kalamazoo Michigan USA

**Keywords:** brown‐headed cowbirds, neighboring species, red‐winged blackbird, referential alarm calls, yellow warbler

## Abstract

In functionally referential communication systems, the signaler's message intended for a conspecific receiver may be intercepted and used by a heterospecific eavesdropper for its own benefit. For example, yellow warblers (*Setophaga petechia*) produce seet calls to warn conspecifics of nearby brood parasitic brown‐headed cowbirds (*Molothrus ater*), and red‐winged blackbirds (*Agelaius phoeniceus*) eavesdrop on and recruit to seet calls to mob the brood parasites. Prior work found that warblers nesting closer to blackbirds were less likely to be parasitized, suggesting that blackbirds may even be the target of warbler's seet calls to assist with antiparasitic defense. Here we discovered for the reverse to apply too: blackbirds nesting closer to yellow warblers also experienced lower probability of brood parasitism. Concurrently, we also found that blackbirds nesting closer to other blackbirds also experience lower parasitism rates. Although these are strictly correlational results, they nonetheless suggest that blackbirds are better able to defend their nest against cowbirds when also listening to nearby warblers' referential alarm calls.

## Graphical abstract

Yellow warbler hosts of brood parasitic brown‐headed cowbirds respond to parasites with referential alarm calls. Red‐winged blackbirds respond to these alarm calls with anti‐cowbird defenses. We discovered that nests of blackbirds that are closer to warblers have lower parasitism rates.

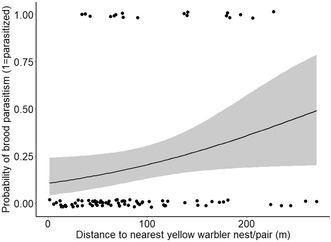

## 1 INTRODUCTION

Animals across diverse lineages use acoustic signals, known as alarm calls, to warn others of nearby threats, such as predators (Hollen & Radford, 2009). Some alarm calls, termed functionally referential alarm calls, refer to specific predators in the environment (e.g., a flying vs. ground predator) and induce escape behaviors in listeners that are adaptive against the specific predator type (e.g., running to cover in response to a flying predator) (Evans et al., 1993). Listeners of referential alarm calls include both intended receivers, such as mates and offspring, as well as unintended eavesdroppers, such as unrelated conspecifics and heterospecifics that could use the information for their own benefit (Gill & Bierema, 2013; Magrath et al., 2015). The use of functionally referential alarm calls for predator detection appears to be widespread, with strong fitness benefits (Blumstein, 1999; Gill & Bierema, 2013).

Predator detection via referential alarm calls occurs in songbirds during the breeding season, for both to engage in active nest defense through mobbing (Feeney & Langmore, 2015; Gill & Sealy, 2003) and to quiet nestlings to avoid nest detection by predators (Haff & Magrath, 2012; Platzen & Magrath, 2005). Predators, however, are not the only threat to nesting songbirds. In North America, over 200 species are parasitized by the brown‐headed cowbird (*Molothrus ater*, hereafter “cowbird”), a brood parasite that forgoes building its own nest and instead deposits eggs into the nests of other species (called “hosts”; Davies, 2010, Figure 1). For hosts, raising a cowbird nestling is costly (Hauber, 2003) and, in response, many species display aggression, both vocal and physical, toward cowbirds to prevent them from parasitizing their nests in the first place (called “frontline defenses”; Feeney & Langmore, 2015; Welbergen & Davies, 2009). The yellow warbler (*Setophaga petechia*, hereafter “warbler”), a common host species of the cowbird (sensu Winfree, 1999), is the only known host of cowbirds to possess an antiparasitic referential alarm call that signals the presence of nearby cowbirds (Gill & Sealy, 2004). The referential “seet” call is produced by these warblers in response to cowbird cues (such as cowbird vocalizations or model presentations), and females that hear this call react as they would to a cowbird, by returning to and positioning themselves upon their nests to prevent the cowbird from inspecting and/or parasitizing the nest (Gill & Sealy, 2004). Recently we discovered that the red‐winged blackbird (*Agelaius phoeniceus*, hereafter “blackbird”), another cowbird host species that often nests near warbler nests, eavesdrops on seet calls and responds with similar aggression toward both playbacks of warbler's seet calls and playbacks of cowbird chatter calls (Lawson et al., 2020; Lawson, Enos, Gill, & Hauber, 2021).

**FIGURE 1:**
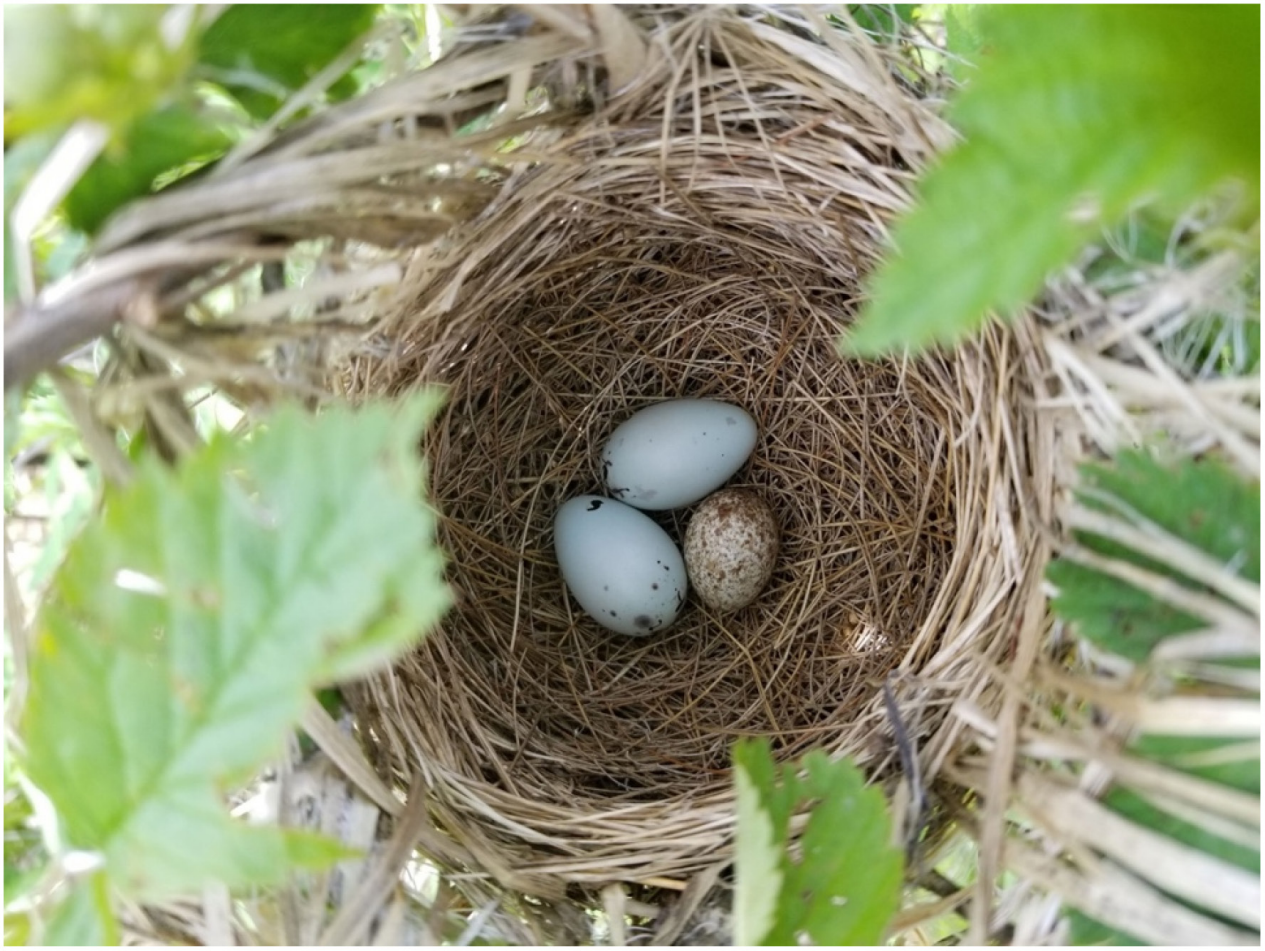
A red‐winged blackbird nest with two eggs that has been parasitized by a brown‐headed cowbird egg (bottom). Photo credit: S. Lawson.

Beyond blackbirds eavesdropping on warbler seet calls, the nesting association between these species leads to documented fitness benefits, at least for warblers; specifically, warblers nesting in closer proximity to blackbird nests experience lower brood parasitism rates compared to those nesting further away (Clark & Robertson, 1979), a benefit attributed to the strong vocal and physical aggression blackbirds exhibit toward cowbirds that approach blackbird nests (e.g. Yasukawa et al., 2016). Blackbird nests may also experience lower brood parasitism risk when in closer proximity to warbler nests, especially considering that blackbirds are already known to receive nest‐defense benefits when breeding in dense conspecific aggregations (e.g., Picman et al., 1988; Weatherhead & Sommerer, 2001; sensu Massoni & Reboreda, 2001). Since the probability of blackbirds detecting cowbirds is enhanced by detecting and responding to nearby warblers' referential seet call (Lawson et al., 2020), we predict that brood parasitism risk will be lower at blackbird nests closer to warbler nests. We also set out to assess whether breeding proximity to another blackbird nest/territory is a negative predictor of cowbird parasitism at our study sites.

## 2 METHODS

Using systematically collected nest content and location data on warbler and blackbird nests gathered from May through July across three field seasons (2019–2021) in central Illinois, USA, we analyzed whether blackbirds that placed nests closer to actively breeding warbler or blackbird pairs experienced lower probabilities of brood parasitism. Detailed study site descriptions, nest searching/monitoring methodology, and how we determined warbler breeding status are described in detail in Lawson et al. (2020); Lawson, Enos, Mendes, et al. (2021). Briefly, we searched six sites in Champaign and Vermilion County two to three times a week for blackbird and warbler nests. For each nest, we noted parasitism status (parasitized vs. nonparasitized, binary status) and took location points for nest sites using GPS units to 3 m accuracy (Garmin model eTrex 10).

For breeding warbler pairs whose nests we could not find, we took a location point for one of the male's singing posts within the territory and used these points as proxies for active warbler nest locations. We determined breeding status by spot‐mapping pairs several times a week and noting behavioral cues strongly associated with an active nest present on territory (see Lawson, Enos, Mendes, et al., 2021 for a detailed description of warbler breeding status methods). For blackbirds, we only used known nests as location points, as we needed to include the parasitism status for each nest in our analyses.

We used ArcGIS (ver. 10.8.1; Redlands, 2022) to calculate the distance (m) between each blackbird nest and the closest warbler and blackbird nest or breeding pair location, within each year of the study. For analyses, we only used data from sites with both warbler and blackbird nests within a given year (2019, *n* = 6 sites; 2020, *n* = 4; 2021, *n* = 5). Blackbirds are facultatively polygynous and can have multiple active breeding nests in their territory at a time (Searcy & Yasukawa, 2014), which introduces biological pseudoreplication as an issue. Thus, we focused on whether the male blackbird's territory experienced any parasitism in any of his nests, by assigning male territories as “non‐parasitized” if no nests were parasitized or “parasitized” if at least one nest was parasitized. Distance to nearest warbler nest/pair or blackbird nest was averaged for all nests on a male blackbird's territory to produce a single distance metric to warbler and to blackbird nest per blackbird territory. Distances between blackbird nests and nearest warbler nest/pair ranged from 1.69 m to 2581.0 m, with most data points (>80%) falling linearly under the 300 m range. Therefore, for statistical purposes, we only included warbler nests and blackbird territories within 300 m of each other to minimize skewness of data and to increase model fit.

We ran a binary logistic regression model to test if distance to nearest warbler nest/pair significantly predicted the probability of brood parasitism on blackbird territories, with distance to the nearest nest as a fixed effect. We did not include year and/or site as fixed or random effects due to small sample sizes. No other variables were included in the model such as the blackbird or warbler nests' breeding stage (sensu Massoni & Reboreda, 2001). We also ran a second binary logistic regression model, with averaged distance to the nearest blackbird nest as a fixed effect instead of warbler nests. We ran all the analyses using R version 4.2.2 and the lme4 package (Bates et al., 2014) and set α = 0.05.

## 3 RESULTS

**FIGURE 2:**
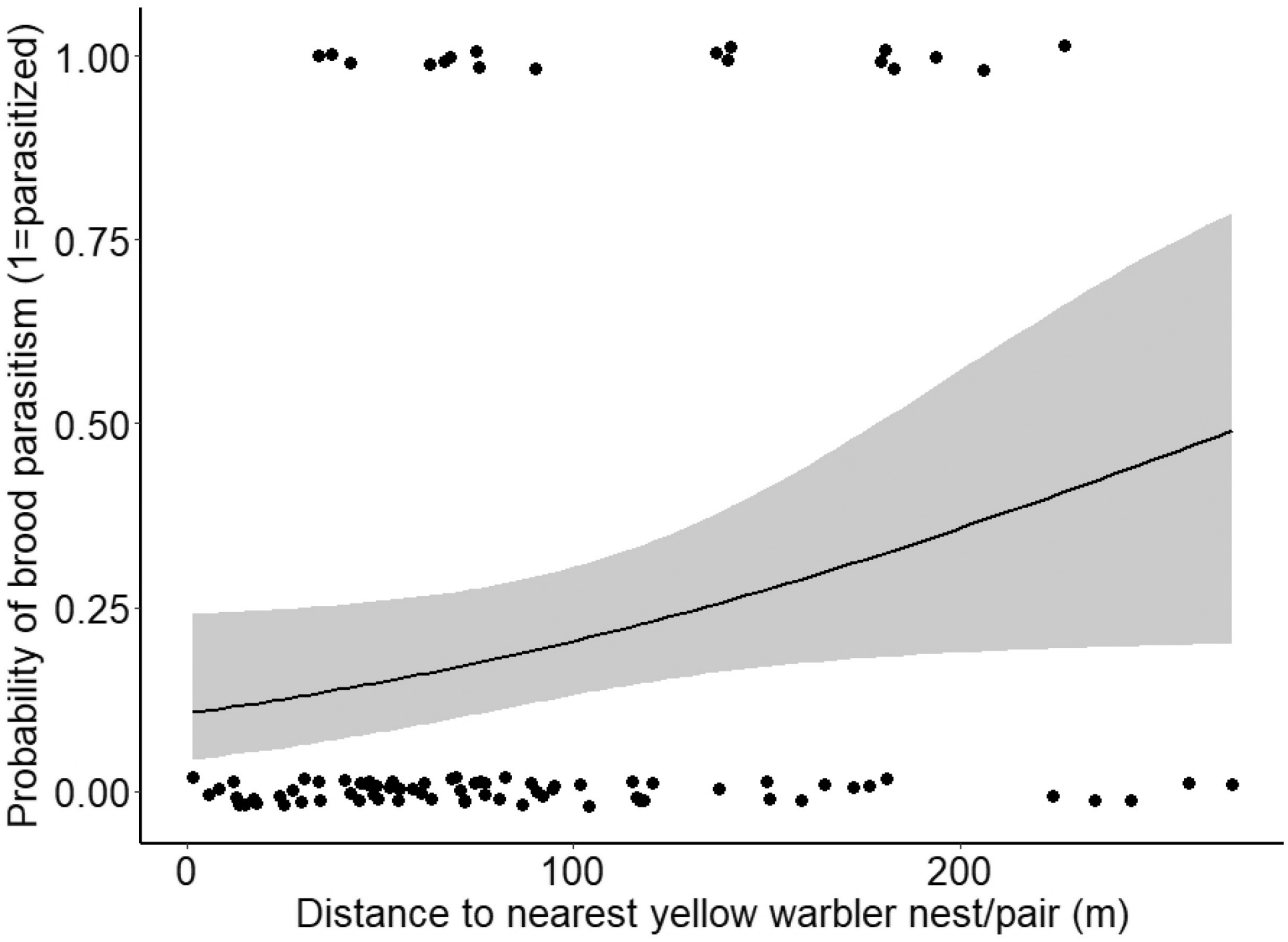
Binary logistic regression of probability of brood parasitism on blackbird territories in relation to distance (m) to nearest yellow warbler nest/pair (distances averaged per territory). Black circles represent blackbird territory locations; shaded area represents 95% confidence limits.

**FIGURE 3:**
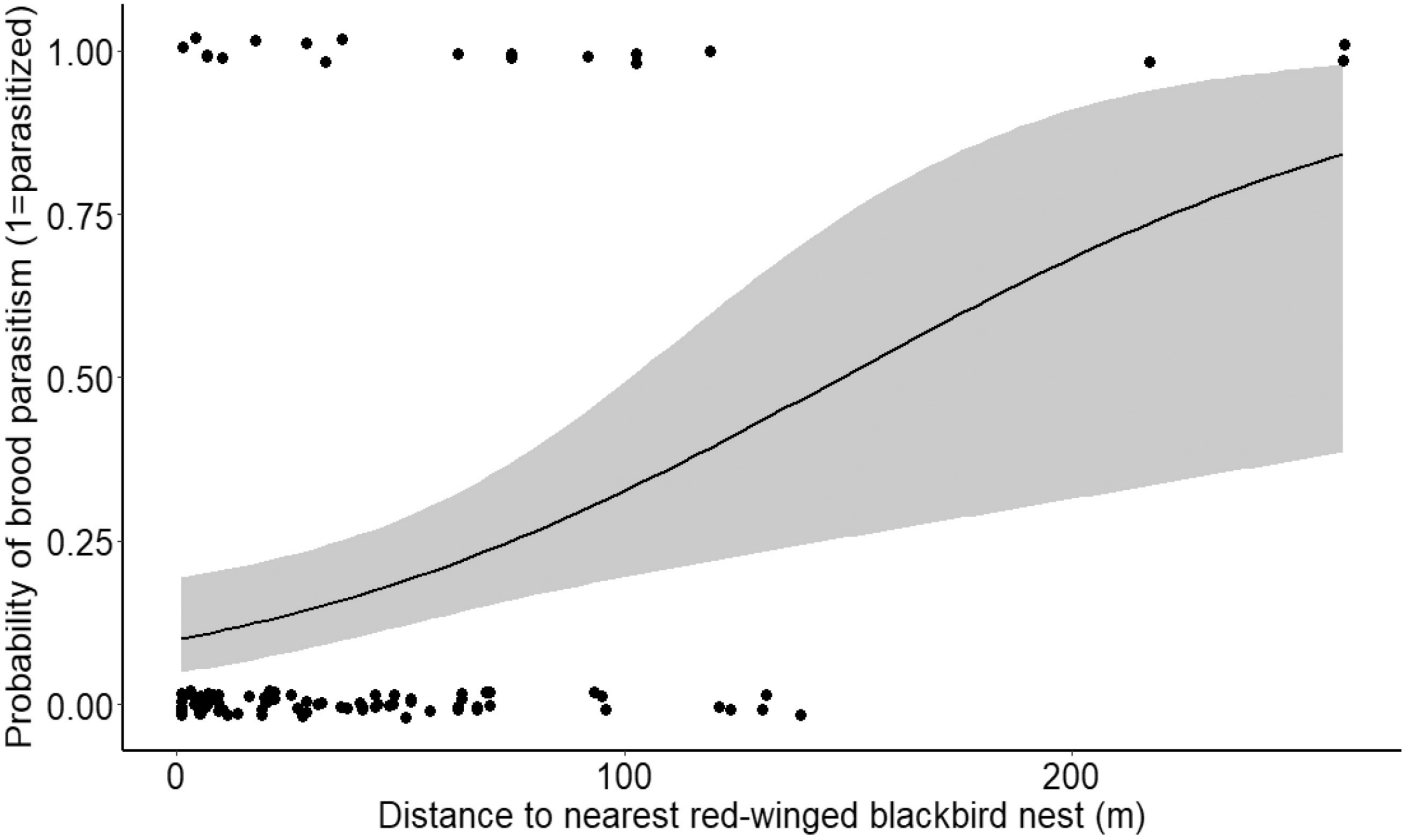
Binary logistic regression of probability of brood parasitism on blackbird territories in relation to distance (m) to nearest blackbird nest (nest distances averaged per territory). Black circles represent blackbird nest locations; shaded area represents 95% confidence limits.

In total, 200 blackbird nests were found across 108 male blackbird territories (average 1.85 nests per territory), as well as 62 warbler nests and 26 breeding pairs locations. Our <300 m distance limit brought our final sample to 88 blackbird territories. We found that distance to nearest warbler nest/pair significantly and positively influenced the probability of brood parasitism on blackbird territories (*F*_1,88_ = 3.95, *p* = .049). Accordingly, blackbirds that nested further from warbler neighbors were more likely to be parasitized compared to those nesting closer to warblers (Figure 2). Likewise, the distance to the nearest blackbird nest also had a significant and positive effect on probability of brood parasitism (*F*_1,97_ = 9.71, *p* = .002; Figure 3).

## 4 DISCUSSION

Our novel, albeit strictly correlational, results here suggest that blackbirds nesting closer to warblers experience a lower risk of brood parasitism. Conspecific nest distance also positively influenced brood parasitism risk, largely confirming previously existing studies on the benefits of conspecific clustering for nest defense in blackbirds (Picman et al., 1981; Weatherhead & Sommerer, 2001). The mechanism by which interspecific benefits act was not directly tested, but the lower parasitism risk may be due to better detection of the warblers' referential alarm calls that could inform blackbirds about cowbird presence on their own territories. Indeed, our previous research showed that blackbirds eavesdrop on and respond to warbler seet calls with their own nest defense behaviors to prevent brood parasitism (Lawson et al., 2020) and that expression of defensive behaviors varied with current brood parasitism risk on the blackbirds' nests within their own territories (Lawson, Enos, Gill, & Hauber, 2021). Taken together with our previous research, we suggest that blackbirds use warbler seet calls produced by nearby neighbors as an early warning system to improve vigilance as well as defense at their own nests.

Given our conspecific nest distance results, we cannot rule out that dilution effects or communal mobbing from surrounding blackbirds also contributed to the reduced brood parasitism risk detected here (Massoni & Reboreda, 2001; Picman et al., 1981; Weatherhead & Sommerer, 2001), either independently or interactively with heterospecific defense. As such, further research is needed to assess whether the decrease in brood parasitism risk leads to additional fitness outcomes, such as greater fledging success and/or successful recruitment of blackbird offspring into the breeding population. If so, there could be a mutualistic relationship between yellow warblers and red‐winged blackbirds, as warblers also experience decreased brood parasitism when nesting closer to blackbirds (Clark & Robertson, 1979). It may even be possible that warblers use seet calls to recruit blackbirds to engage in group defense against the parasitism threat nearby (sensu Kelly et al., 2019). The simplest first step to testing this “mutualistic nest neighbor” hypothesis is to determine blackbird and warbler preferences for establishing breeding territories near one another in the spring. In Illinois, blackbird males establish territories before warblers arrive in the spring, pointing to the testable prediction that warblers are the ones to preferentially settle closer to blackbirds, rather than vice versa.

Other host co‐occurring species may need to be considered to test the mutualistic nest neighbor hypothesis, which we did neither here nor in previous studies (Lawson et al., 2020; Lawson, Enos, Gill, & Hauber, 2021). If no other host species responds aggressively to warbler seet calls or benefit from nesting near warblers, then this would provide some more evidence of a mutualistic relationship between red‐winged blackbirds and yellow warblers specifically.

## AUTHOR CONTRIBUTIONS

**Shelby L. Lawson:** Conceptualization (equal); data curation (lead); formal analysis (lead); investigation (equal); methodology (equal); visualization (lead); writing – original draft (lead). **Janice K. Enos:** Conceptualization (equal); investigation (equal); methodology (equal); writing – review and editing (supporting). **Sharon A. Gill:** Funding acquisition (equal); resources (equal); supervision (equal); writing – review and editing (supporting). **Mark E. Hauber:** Conceptualization (equal); funding acquisition (equal); project administration (equal); resources (equal); supervision (equal); writing – original draft (equal).

## Data Availability

The original contributions presented in the study along with the R data code can be found at https://figshare.com/s/f4cad71f2f1db2868220.
